# Rotating Stall Induced Non-Synchronous Blade Vibration Analysis for an Unshrouded Industrial Centrifugal Compressor

**DOI:** 10.3390/s19224995

**Published:** 2019-11-16

**Authors:** Xinwei Zhao, Qiang Zhou, Shuhua Yang, Hongkun Li

**Affiliations:** 1 School of Mechanical Engineering, Dalian University of Technology, No. 2 Linggong Road, Dalian 116024, China; xinweizhao@mail.dlut.edu.cn (X.Z.); zhouqdut@mail.dlut.edu.cn (Q.Z.); 2 Shenyang Blower Works Group, No.16 Development Road, Shenyang 110869, China; yangshuhua@shengu.com.cn

**Keywords:** rotating stall, non-synchronous blade vibration, blade tip timing, centrifugal compressor

## Abstract

Rotating stall limits the operating range and stability of the centrifugal compressor and has a significant impact on the lifetime of the impeller blade. This paper investigates the relationship between stall pressure wave and its induced non-synchronous blade vibration, which will be meaningful for stall resonance avoidance at the early design phase. A rotating disc under a time-space varying load condition is first modeled to understand the physics behind stall-induced vibration. Then, experimental work is conducted to verify the model and reveal the mechanism of stall cells evolution process within flow passage and how blade vibrates when suffering such aerodynamic load. The casing mounted pressure sensors are used to capture the low-frequency pressure wave. Strain gauges and tip timing sensors are utilized to monitor the blade vibration. Based on circumferentially distributed pressure sensors and stall parameters identification method, a five stall cells mode is found in this compressor test rig and successfully correlates with the blade non-synchronous vibration. Furthermore, with the help of tip timing measurement, all blades vibration is also evaluated under different operating mass flow rate. Analysis results verify that the proposed model can show the blade forced vibration under stall flow condition. The overall approach presented in this paper is also important for stall vibration and resonance free design with effective experimental verification.

## 1. Introduction

Centrifugal compressors have the advantages of high single-stage pressure ratio, wide working range, and compact structure. They are highly valued and broadly applied, such as turbochargers in automotive engines, the processing of natural gas, aerospace, gas turbine engines and so on [1]. With the improvement of industrial requirements and aerodynamic design, the structural integrity of rotating impeller meets great challenges. During the operation of the compressor, the blade vibrates due to mechanical parts and unsteady aerodynamic loads. The unsteady aerodynamic load is inherently and can cause large blade vibration and even high cycle fatigue (HCF) failure. The most common flow-induced vibration is due to rotor-stator interaction. The variable inlet guide vanes (VIGVs) and diffuser vanes (DVs) are two main typical excitation sources in centrifugal compressor stage. However, the impeller resonance caused by these exciting sources can mostly be avoided by utilizing Campbell diagram during the design phase [2]. Currently, the most challenging problems occur at off-design conditions. Blade failures can also occur and are caused by rotating stall effects as Haupt et al. experimentally found [3]. The rotating stall cells are also the possible blade excitation source. It is a localized phenomenon and the compressor can still give acceptable aerodynamic performance. However, it will result in circumferential non-uniform and periodic pressure pulsation (stall mode) in blade rows and form a transient rotating stall dominated exciting force applying on the entire impeller. In some specific cases, the impeller modes may be excited and resonance occurs, which is undesirable for blade structure. The unstable operating behavior of rotating stall and induced blade forced response mechanism are not yet fully understood [4]. The impact of the rotating cells on blade vibration amplitude and stress level is also unknown. It is still a key aspect in the academic and industrial fields of turbomachinery.

A great number of numerical and experimental studies have been conducted to reveal the mechanism of rotating stall [4]. However, compared to the axial compressor [5,6,7,8], researches about rotating stall happened in centrifugal compressors start later [9,10]. Abdelhamid et al. [11,12] investigated the effects of vaneless diffusers with different geometries on rotating stall. Frequency components were analyzed in detail and they found that the stall frequency was dependent on the diffuser radius ratio and the complex coupling between the impeller and the diffuser. In addition to Abdelhamid’s work, many experimental tests investigating the influence of different geometrical configurations were also conducted by Ferrara [13]. These results are plentiful and give a more detailed benchmark for a large number of geometry configurations. Fujisawa et al. [14,15] combined experimental and numerical methods to study the unsteady flow phenomenon of rotating stall in diffuser passage. Numerical results showed a vortex generated at the diffuser leading-edge is the main cause of the stall. Stall occurrence in centrifugal compressor with vaned and vaneless diffusers has different features. In addition, both the impeller and the diffuser, even the return channel [16], will have rotating stall and origin differs. All of these raise the uncertainty and randomness of the rotating stall happened in the centrifugal compressor.

The literature mentioned above contributes to the causes and evolution of the rotating stall from an aerodynamic perspective. Rotating stall induced non-synchronous vibration (NSV) and resonance should be paid more attention. Currently, only a small part of the research work focuses on the stall induced vibration mechanisms and quantitative assessment of real applied industrial compressors. J. Chen [17] used dynamic pressure transducers and strain gauges to record the unsteady pressure fields and blade vibration. Two different stall modes were reported in this work. Although the flow mechanism of the rotating stall was explained, the blade vibration was still not analyzed in detail. Seidel et al. [18,19] observed large amplitudes of vibration occurred in centrifugal compressor impeller. Identified seven stall cells which induce dangerous blade vibration was also reported in recent work by Jenny [20] using the IGV sweep method. Higher harmonics of rotating stall disturbance in Ref. [21] was also found to be the cause of blade vibration. Vahdati et al. [22] conducted numerical simulation to investigate the axial compressor stall process observed in the experiments. The predicted stall cells number was quite close to the identified results. The numerical method they used provided a promising approach to study the stall and blade vibration. Others are concerned about rotor vibration caused by rotating stall, such as Reference [23], which gave a discussion centered on forced vibration of rotors depending on the vibration characteristics. Moreover, Ferrari et al. [24,25] put great efforts to experimentally quantify the force coming from rotating stall. Identified amplitude and frequency of the external force acting on the impeller is further included in the rotodynamic model of the test rig to get more accurate results.

Currently, the stall imposed aerodynamic forcing function is not theoretically built up. The general resonance condition of impeller blade under rotating stall excitation is also not modeled and deduced, which will be highly meaningful to understand the physics. In addition, the impact of the stall cells on all blade vibration is not quantitatively accessed, which is the basis for aeroelastic design of impeller blade. Related results are still far from providing a complete explanation of this phenomenon. Mechanisms behind this complex fluid-structure coupling phenomenon should also be interpreted more in detail. Based on the above aims, the remainder of the paper is organized as follows to achieve this goal. In Section 2, the rotating stall forcing function traveling around the impeller is built up. Then, the asynchronous traveling excitation of the impeller structure is modeled. The resonance condition is deduced using steady part of the blade forced response function. Based on the theoretical model, Section 3 introduces the test rig and experimental method in order to acquire the substantial data which is needed to verify the model and understand the rotating stall phenomenon. In Section 4, experimental results are analyzed in detail. The identified rotating stall pattern is further parameterized and correlated with the blade non-synchronous vibration. Finally, all blade vibration is monitored and quantified with a large amount of tip timing data for structure assessment purpose. These vibration data will also be useful for aeroelastic design of impeller blade under stall condition. At last, the conclusions are given in Section 5.

## 2. Rotating Stall Identification and Resonance Condition for Impeller

### 2.1. Parameter Characterization of Rotating Stall

**Figure 1:**
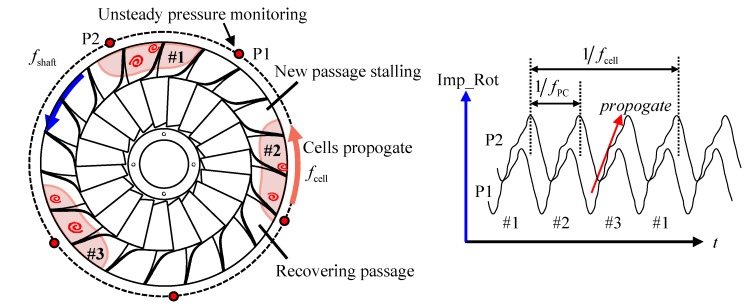
Dynamic characteristics of rotating stall in a centrifugal compressor impeller.

According to its real behavior recorded by the pressure sensor, the rotating stall can be assumed to have a nearly uniform distribution after the stall cells stably formed in the flow passage. Figure 1 gives a brief picture of typical rotating stall happened in a centrifugal compressor. Considering rotating stall will also occur in the vaned diffuser, these stall cells are only drawn for illustration and used to describe the stall region presented circumferentially. The rotating stall state can be uniquely defined by the number of stall cells ${\mathrm{NB}}_{C}$, their rotation speed $f_{\mathrm{cell}}$ and propagate direction relative to the impeller. These parameters can be identified by proper interpretation of the pressure fields within the compressor during rotating stall.

Since these stall cells are self-similar and cannot be directly identified by the FFT spectrum. Several pressure transducers should be used to monitor circumferential pressure fluctuating and combined time-frequency analysis method is needed. Assume that there are 2 monitoring points in the circumferential direction, namely P1 and P2. For the stall cell #1, it will pass through P1 and P2 successively, causing the time-delay characteristics of the signals collected in the two sensors. The time delay and cell propagation are also shown in the right picture of Figure 1. The frequency $f_{\mathrm{PC}}$ detected by the unsteady pressure sensor mounted on the casing corresponds to the product of the number of stall cells ${\mathrm{NB}}_{C}$ and the rotating frequency $f_{\mathrm{cell}}$ of the individual stall cell in stationary coordinate

(1) $$f_{\mathrm{PC}}={\mathrm{NB}}_{C}\cdot f_{\mathrm{cell}}$$

### 2.2. Identification of Stall Induced Impeller Vibration and Resonance

**Figure 2:**
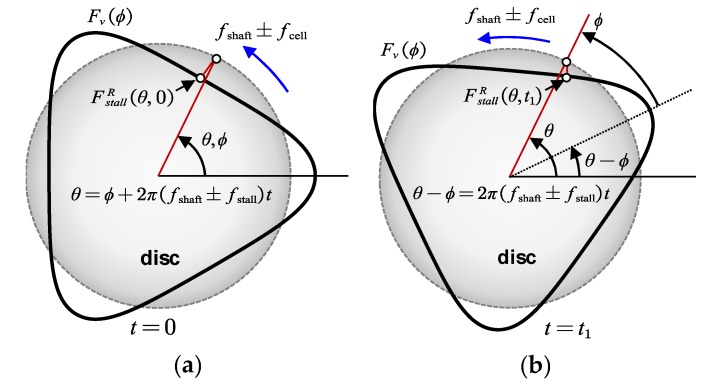
Rotating stall induced impeller forced vibration model based on simplified disc vibration behavior at the time t=0 (**a**) and $t=t_{1}$ (**b**) in RCS.

Since the rotating stall is related to flow separation, the pressure loading is distorted circumferentially and can be described as a rotating wave arising and decaying intermittently. The induced non-uniform force is approximated as a sinusoidal signal here and other forms can refer to Ref. [26]. Corresponding aerodynamic force (thick black line in Figure 2) is then built up in rotating coordinate system (RCS). In RCS, the impeller is static while the aerodynamic force is traveling around it. The relative moving speed between impeller and stall cells corresponds to the frequency difference between shaft frequency $f_{\mathrm{shaft}}$ and individual cell rotating frequency $f_{\mathrm{cell}}$. If the stall cells rotate in the same direction as the impeller, the exciting frequency will be $f_{\mathrm{shaft}}-f_{\mathrm{cell}}$, otherwise, the value is $f_{\mathrm{shaft}}+f_{\mathrm{cell}}$. It can be found through simple analysis. If the stall cells rotate in the same direction, the impeller must rotate more in order to form a periodic load (Stall cells also move a certain distance in the same direction during the impeller rotation), thus making this period longer than shaft rotating period $1/f_{\mathrm{shaft}}$. Relationship between pressure pulsation frequency of rotating stall and blade vibration can be calculated by the following expression

(2) $$f_{e}=n_{e}\cdot {\mathrm{NB}}_{C}\left(f_{\mathrm{shaft}}\pm f_{\mathrm{cell}}\right)=n_{e}\cdot \left({\mathrm{NB}}_{C}\cdot f_{\mathrm{shaft}}\pm f_{\mathrm{PC}}\right),\;n_{e}\in {\mathbb{Z}}_{+}$$

where $n_{e}$ is the harmonic index, $f_{\mathrm{shaft}}$ is the rotating speed of impeller, and $f_{e}$ is the frequency of the exciting force.

Equation (2) gives the relationship between rotating stall parameters and non-integer engine order excitation. However, it is still independent of the impeller vibration mode and cannot explain when the impeller will resonate and which mode is excited. Thus, forced response modal should be further built up so that the resonance conditions for impeller under rotating stall condition can be derived. The pressure pulsation produced by the rotating stall is a periodic time-varying function. In stationary coordinate system (SCS), the aerodynamic force $F_{stall}^{s}\left(\phi \right)$ along the circumference of the rotating impeller can be described as

(3) $$\left\{ \begin{array}{l} F_{stall}^{s}\left(\phi ,t\right)=F_{v}\left(\phi \right)\cos\left(2\pi {\mathrm{NB}}_{c}\cdot f_{\mathrm{cell}}t\right),\;\phi \in \left[0,2\pi \right]\\ F_{v}\left(\phi \right)=\sum_{k}\left(a_{k}\cos\left[k\cdot {\mathrm{NB}}_{c}\cdot \phi \right]+b_{k}\sin\left[k\cdot {\mathrm{NB}}_{c}\cdot \phi \right]\right) \end{array}\right.$$

where $F_{v}\left(\phi \right)$ corresponds to the distribution of force, $a_{k}$ and $b_{k}$ are the corresponding Fourier coefficients. ${\mathrm{NB}}_{c}$ and $f_{\mathrm{cell}}$ are rotating stall parameters mentioned above. Then, the distributed force can be further transformed into rotating coordinate

(4) $$\left\{ \begin{array}{l} F_{stall}^{R}\left(\theta ,t\right)=F_{v}\left(\phi \right)\cos\left[n_{e}\cdot {\mathrm{NB}}_{c}\cdot \Omega_{stall}(t+t_{\phi })\right]\delta \left[\theta -\Omega_{stall}t-\phi \right]\\ t_{\phi }=\phi /\Omega_{stall},\;\Omega_{stall}=2\pi \left(f_{\mathrm{shaft}}\pm f_{\mathrm{cell}}\right) \end{array}\right.$$

where δ[⋅] is the Dirac delta function, θ is the angle measured in impeller rotating coordinate, $t_{\phi }$ is a time delay due to the phase shift between ${F_{stall}^{R}\left(\theta ,t\right)|}_{t=0}$ and $F_{v}\left(\phi \right)$. Relationship between $F_{v}\left(\phi \right)$ and $F_{stall}^{R}\left(\theta ,t\right)$ at two different times is drawn in Figure 2a,b. The thick black line in Figure 2 corresponds to the aerodynamic force of the rotating stall. The circular disc corresponds to the impeller. The dynamical behavior of the distributed force described by Equation (4) has been well presented. It shows the relative rotation of the stall cells on the impeller. Furthermore, with such circumferential moving, the phase between the impeller and aerodynamic load changes accordingly.

The impeller eigenmodes with ND-th nodal diameters of blade vibration can be defined as sine mode with the phase θ in RCS

(5) Φ(θ)=sinNDθ  (ND=0,1,2…,N−1/2  or  N/2)

which is the modeling and analysis treatment method for vibration of mechanical structures with cyclic symmetry property. And, the mistuning of the real impeller is ignored here.

So far, the traveling excitation force corresponding to typical rotating stall condition has been derived. And, the impeller mode is also characterized into different waves. Qualitative analysis of the forced response of a rotating disc can be derived step by step according to Ref. [27]. The generalized force Q(t) for the ND nodal diameter mode can be first acquired by

(6) $$\begin{array}{l} Q\left(t\right)=\int_{0}^{2\pi }F_{stall}^{R}\left(\theta ,t\right)\Phi \left(\theta \right)\;d\theta \\ \;\;\;=\int_{0}^{2\pi }F_{v}\left(\phi \right)\cos\left[n_{e}\cdot {\mathrm{NB}}_{c}\cdot \Omega_{stall}(t+t_{\phi })\right]\delta \left[\theta -\Omega_{stall}t-\phi \right]\sin\mathrm{ND}\theta \;d\theta \;\\ \;\;\;=F_{v}\left(\phi \right)\cos\left[n_{e}\cdot {\mathrm{NB}}_{c}\cdot \Omega_{stall}(t+t_{\phi })\right]\sin\mathrm{ND}(\Omega_{stall}t+\phi )\; \end{array}$$

Based on Equation (6), convolution integral is then used to acquire normal response on the disc for this lightly-damped system, that is

(7) $$x_{\phi }\left(\phi ,\theta ,t\right)=\frac{1}{m_{ND}\omega_{ND}}\int_{0}^{t}Q\left(\tau \right)\sin\omega_{ND}(t-\tau )\;d\tau$$

where $m_{ND}$ and $\omega_{ND}$ are the corresponding modal mass and damped natural frequency. Substituting the generalized force in Equation (6) into the above formula, we can get the disc normal response

(8) $$\begin{array}{l} x_{\phi }\left(\phi ,\theta ,t\right)=\frac{1}{m_{ND}\omega_{ND}}\int_{0}^{t}F_{v}\left(\phi \right)\cos\left[n_{e}\cdot {\mathrm{NB}}_{c}\cdot \Omega_{stall}(\tau +t_{\phi })\right]\sin\mathrm{ND}(\Omega_{stall}\tau +\phi )\;\sin\omega_{ND}(t-\tau )\;d\tau \\ =\frac{F_{v}\left(\phi \right)}{4m_{ND}\omega_{ND}}\left(\begin{array}{l} -\frac{\sin\left(-\phi {\mathrm{NB}}_{C}n_{e}-\omega_{ND}t+\mathrm{ND}\phi \right)}{\Omega_{stall}\left(\mathrm{ND}-{\mathrm{NB}}_{C}n_{e}\right)+\omega_{ND}}-\frac{\sin\left(-\phi {\mathrm{NB}}_{C}n_{e}+\omega_{ND}t+\mathrm{ND}\phi \right)}{\Omega_{stall}\left({\mathrm{NB}}_{C}n_{e}-\mathrm{ND}\right)+\omega_{ND}}\\ -\frac{\sin\left(\phi {\mathrm{NB}}_{C}n_{e}-\omega_{ND}t+\mathrm{ND}\phi \right)}{\Omega_{stall}\left({\mathrm{NB}}_{C}n_{e}+\mathrm{ND}\right)+\omega_{ND}}+\frac{\sin\left({\mathrm{NB}}_{C}n_{e}\left(\Omega_{stall}t+\phi \right)+\mathrm{ND}\left(\Omega_{stall}t+\phi \right)\right)}{\Omega_{stall}\left({\mathrm{NB}}_{C}n_{e}+\mathrm{ND}\right)+\omega_{ND}}\\ +\frac{2\omega_{ND}}{\omega_{ND}^{2}-\Omega_{stall}^{2}{\left(\mathrm{ND}-{\mathrm{NB}}_{C}n_{e}\right)}^{2}}\sin\left[\mathrm{ND}\left(\Omega_{stall}t+\phi \right)-{\mathrm{NB}}_{C}n_{e}\left(\Omega_{stall}t+\phi \right)\right]\\ +\frac{\sin\left({\mathrm{NB}}_{C}n_{e}\left(\Omega_{stall}t+\phi \right)+\mathrm{ND}\left(\Omega_{stall}t+\phi \right)\right)}{\omega_{ND}-\Omega_{stall}\left({\mathrm{NB}}_{C}n_{e}+\mathrm{ND}\right)}-\frac{\sin\left(\phi {\mathrm{NB}}_{C}n_{e}+\omega_{ND}t+\mathrm{ND}\phi \right)}{\omega_{ND}-\Omega_{stall}\left({\mathrm{NB}}_{C}n_{e}+\mathrm{ND}\right)} \end{array}\right) \end{array}$$

In the preceding equation, terms with $\omega_{ND}t$ correspond to the transient response of the disc and are assumed to decay over time since damping is considered. Only terms with $\Omega_{stall}t$ are of interest. Finally, the steady-state response of the disc under rotating stall disturbance has the following form

(9) $$x_{\phi }\left(\phi ,\theta ,t\right)=\frac{F_{v}\left(\phi \right)}{2m_{ND}}\left(\frac{\sin\left[\left(\mathrm{ND}-n_{e}{\mathrm{NB}}_{c}\right)\left(\phi +\Omega_{stall}t\right)\right]}{\omega_{ND}^{2}-{\left(\mathrm{ND}-n_{e}{\mathrm{NB}}_{c}\right)}^{2}\Omega_{stall}^{2}}+\frac{\sin\left[\left(\mathrm{ND}+n_{e}{\mathrm{NB}}_{c}\right)\left(\phi +\Omega_{stall}t\right)\right]}{\omega_{ND}^{2}-{\left(\mathrm{ND}+n_{e}{\mathrm{NB}}_{c}\right)}^{2}\Omega_{stall}^{2}}\right)$$

The response over the entire pressure distribution should be further integrated by

(10) $$x(\theta ,t)=\int_{0}^{2\pi }x_{\phi }\left(\phi ,\theta ,t\right)\;d\phi$$

Combined with $F_{v}\left(\phi \right)$ and the integral properties of trigonometric functions, two cases can be identified in order to have x(θ,t)≠0. First, for the case of $k{\mathrm{NB}}_{c}=\left|\mathrm{ND}-n_{e}{\mathrm{NB}}_{c}\right|$, it follows from Equation (10) that

(11) $$x^{C1}(\theta ,t)=\frac{\pi }{2m_{ND}A_{1}}\left(a_{k}\sin\left[\left(\mathrm{ND}-n_{e}{\mathrm{NB}}_{c}\right)\Omega_{stall}t\right]+b_{k}\cos\left[\left(\mathrm{ND}-n_{e}{\mathrm{NB}}_{c}\right)\Omega_{stall}t\right]\right)$$

Second, for the case of $k{\mathrm{NB}}_{c}=\mathrm{ND}+n_{e}{\mathrm{NB}}_{c}$,

(12) $$x^{C2}(\theta ,t)=\frac{\pi }{2m_{ND}A_{2}}\left(a_{k}\sin\left[\left(\mathrm{ND}+n_{e}{\mathrm{NB}}_{c}\right)\Omega_{stall}t\right]+b_{k}\cos\left[\left(\mathrm{ND}+n_{e}{\mathrm{NB}}_{c}\right)\Omega_{stall}t\right]\right)$$

$A_{1}$ and $A_{2}$ in Equations (11) and (12) have the following form

(13) $$\left\{ \begin{array}{l} A_{1}=\omega_{ND}^{2}-{\left(\mathrm{ND}-n_{e}{\mathrm{NB}}_{c}\right)}^{2}\Omega_{stall}^{2}\\ A_{2}=\omega_{ND}^{2}-{\left(\mathrm{ND}+n_{e}{\mathrm{NB}}_{c}\right)}^{2}\Omega_{stall}^{2} \end{array}\right.$$

The discussion of these two cases is due to the different directions of rotation of the stall cells. Due to the different propagation nature, Equations (11)–(13) is treated separately. These equations can be combined to describe the steady response of the impeller structure under different stall conditions. It is obvious that the resonance will occur if $A_{1}$ and $A_{2}$ in Equations (11) and (12) become zero. Thus, two different frequency relationship between impeller mode and dynamic stall mode can be built up based on the cell propagation direction

(14) $$\omega_{ND}=\left\{ \begin{array}{l} \left|n_{e}{\mathrm{NB}}_{c}-\mathrm{ND}\right|\Omega_{stall},\;\;k{\mathrm{NB}}_{c}=\left|n_{e}{\mathrm{NB}}_{c}-\mathrm{ND}\right|\\ \left(n_{e}{\mathrm{NB}}_{c}+\mathrm{ND}\right)\Omega_{stall},\;\;k{\mathrm{NB}}_{c}=n_{e}{\mathrm{NB}}_{c}+\mathrm{ND} \end{array}\right.$$

$k{\mathrm{NB}}_{c}=\left|n_{e}{\mathrm{NB}}_{c}-\mathrm{ND}\right|$ and $k{\mathrm{NB}}_{c}=n_{e}{\mathrm{NB}}_{c}+\mathrm{ND}$, which should not be dropped, are two prerequisites of $A_{1}$ and $A_{2}$, respectively. Further, Equation (14) can be simplified as

(15) $$\left\{ \begin{array}{l} \mathrm{ND}=m{\mathrm{NB}}_{c},\;m\in {\mathbb{Z}}_{+}\\ f_{ND}=m{\mathrm{NB}}_{c}\left(f_{\mathrm{shaft}}\pm f_{\mathrm{cell}}\right) \end{array}\right.$$

where $f_{ND}$ is the natural frequency of impeller in Hz. Equation (15) points out that for $\mathrm{ND}={\mathrm{NB}}_{c}$ nodal diameter mode, the impeller resonance will occur if the natural frequency coincides with ${\mathrm{NB}}_{c}\left(f_{\mathrm{shaft}}\pm f_{\mathrm{cell}}\right)$. This is the most common situation happened during a lot of authors’ experimental research [18,19,20,21]. The number of stall cells is very limited, which means excitation frequency will not be very high for industrial centrifugal compressors. In these cases, the first-order vibration mode will be quite close to the excitation frequency and needs to be considered. In addition to the basic disturbance coming from rotating stall, the higher harmonic component will also induce the impeller resonance with higher nodal diameter modes $m{\mathrm{NB}}_{c}$. However, the actual number of nodal diameters needs to be further calculated based on the specific number of blades. Although this situation is not common and very rare, it has also been found to exist by Mischo [21]. Since the circumferential non-uniform load caused by rotating stall with different origins (diffuser stall or impeller stall) is consistent, the derived equation is a general relationship and will be useful for impeller resonance check considering the rotating stall effects.

## 3. Test Facilities and Measurement Procedure

**Figure 3:**
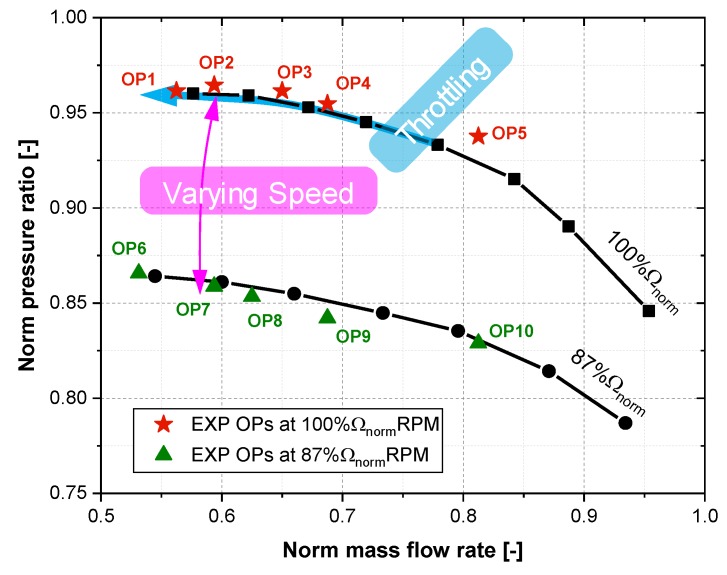
Operation line and experimental measurement scheme of the compressor facility.

The experiments with full-size single-stage centrifugal compressor (SSCC) facility are performed at the Shenyang Blower Works Group Corporation. Numerous experimental studies have been conducted to acquire the knowledge of the compressor blade vibration under rotating stall condition. Since the stall and surge condition will do great harm to the compressor test rig, these operating points are carried out carefully during the experiments. Both transient and quasi-steady operating conditions are tested in detail. Figure 3 presents the operation line of the compressor test rig and shows detailed experimental measurement scheme. The behavior of the compressor test rig near the stall and surge boundary is mostly concerned. The compressor stage is first throttled at $100\%\Omega_{\mathrm{norm}}$ speed to study the stall behavior from aerodynamic point of view. In order to find the rotating stall induced vibration phenomena, speed ramp (continuous varying speed) testing of the compressor at a low mass flow rate is measured. Two operating speeds ($100\%\Omega_{\mathrm{norm}}$ and $87\%\Omega_{\mathrm{norm}}$) are further tested with five selected mass flow rates (OP1-OP10 denoted in Figure 3). These quasi-steady operating conditions are combined to reveal the rotating stall mechanism and quantify the blade vibration. Detailed experimental results and discussion will be given in Section 4. Investigated compressor test rig and different measurement techniques will be elaborated in the following part of Section 3.

### 3.1. Centrifugal Compressor Test Rig

**Figure 4:**
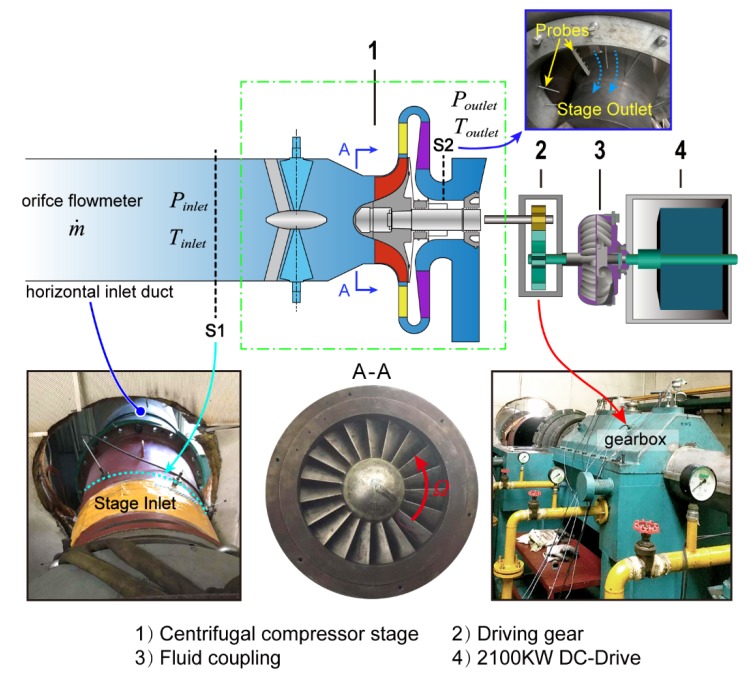
Schematic representation of the experimental test rig.

The structure of the test compressor is shown in Figure 4. It consists of variable inlet guide vanes (VIGVs), an unshrouded backswept centrifugal impeller, a vaned diffuser, and the return channel. The number of blades and the main dimensions of these components are listed in Table 1. IGV blades are fixed in the axial inlet duct and used to deflect the flow in the tangential direction. The designed variable angle range is 40° to 120° (Here, 90° corresponds to the full open of IGV blades). Upstream and downstream of the compressor stage are the horizontal inlet duct and outlet pipe. A 2100 kW Electric motor is installed to drive the compressor with required rotating speeds. The test rig running speed ranges from 500 RPM to 9,000 RPM. A driven gear (drive ratio is 126/43 = 2.93) and a fluid coupling are further used to connect the motor and compressor shaft.

### 3.2. Data Acquisition

#### 3.2.1. Unsteady Static Pressure Measurement

**Figure 5:**
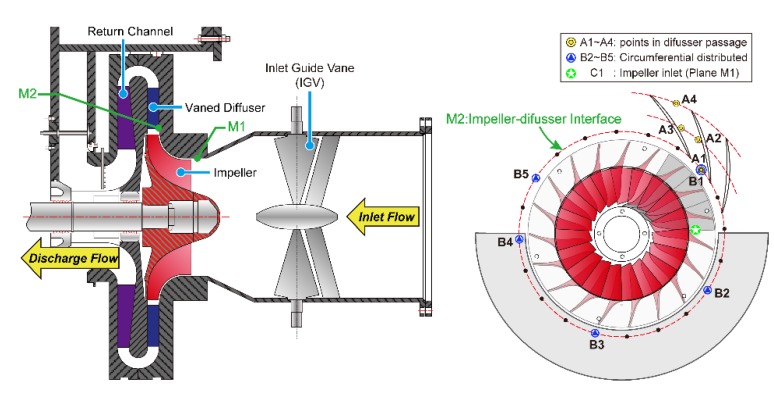
Casing-mounted pressure transducer positions in meridional and axial view.

The unsteady static pressure is measured at several locations within flow passage from compressor inlet to diffuser outlet. Figure 5 presents the meridional and axial view of these transducer positions. In order to determine the flow instability in the streamwise direction, the pressure pulsation at the compressor inlet (M1–C1) and in the diffuser passage (A1–A4) are monitored during the experiment. At the same time, the circumferential pressure pulsation at impeller-diffuser interface (M2) is also monitored for extra 4 points (B2–B5). Monitoring points A1 and B1 are in the same position. Holes used to install the pressure transducer at all these positions are opened at impeller and diffuser shroud casing component. The dynamic probe used here is the Model 106B52 produced by PCB Piezotronics. In order to avoid the disturbance caused by the transducer itself, each sensor is treated carefully and flush-mounted with the casing inner surface. The black dots at measurement plane M2 denotes the same location of A1/B1 at each passage inlet and only two diffuser passages are drawn here for simplicity. The angles in the circumferential direction between B1–B2, B2–B3, and B3–B4 are 72°. Considering non-uniform distribution of the sensors can depict the propagate of rotating stall cells more properly, sensor at location B5 is positioned at the adjacent diffuser passage of B4, such that the angle between B4–B5 is 36 degrees. Upon finishing the installation of these sensors, all signals are transmitted to a signal acquisition card, which is used for multi-channel high-precision measurement. Transient static pressure signals are acquired and recorded in DASP software, provided by Beijing Oriental Vibration and Noise Technology Institute. The sampling rate for the pressure transducers depends on the frequencies of interest. It is set to 20.48 kHz which is enough to resolve the blade passing frequency (BPF) and the rotating stall frequency (RSF).

#### 3.2.2. Strain Gauge and Tip Timing Measurement

**Figure 6:**
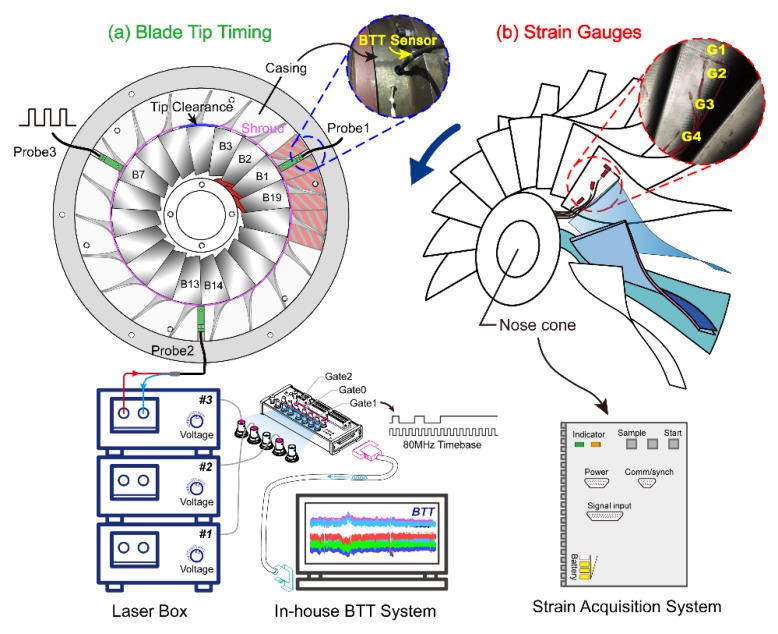
Installation and arrangement of tip timing probes (**a**) on the casing and strain gauges (**b**) on one impeller blade.

In this experiment, the non-synchronous vibration of the blade is monitored by 4 strain gauges in total. The installation position of these strain gauges (G1–G4) is shown in Figure 6b. The strain gauge G3 is positioned at half-span height and near the leading edge of the blade. It is used to capture the blade first bending mode since it is most sensitive to rotating stall disturbance. G1 and G2 are located at the tip of the blade to acquire the response of higher modes considering rotor-stator interactions. G1 is parallel to the blade tip and G2 is perpendicular to G1. At the root of the blade, strain gauge G4 is used to test the strength of the blade. Compact dynamic data acquisition instrument is installed in the nose cone (shown in Figure 6b) of the impeller to continually acquire the signal. A rounded bulb structure is further used to cover the hole. Only the strain gauge is exposed in the airflow environment. Considering the additional mass of the strain acquisition equipment, the balance of the rotor is conducted so that the shaft unbalance vibration is small. Since only one blade vibration is measured in detail by strain gauges, blade tip timing measurement is used to supplement the strain test for detailed vibration quantification. The tip deflection of all blades is measured simultaneously. Figure 6a gives the distribution of the tip timing sensors. The distribution angle between each BTT sensor is 120°. The light source (Red path) of each probe is provided by the laser box. The reflected laser (Blue path) will be transmitted back as each blade passes through the sensor. After receiving the blade triggered pulse, a high-speed counting acquisition card NI6602 is used here to obtain the blade arrival time sequence using 80 MHz internal counter. These data are further combined with the rotating speed and the tip radius to obtain the instantaneous vibration displacement. All pre- and post-processing work of the BTT signal can be finished in the in-house developed measurement system.

## 4. Results and Discussion

### 4.1. Stall Induced Flow Instability Behavior during Throttling Process

**Figure 7:**
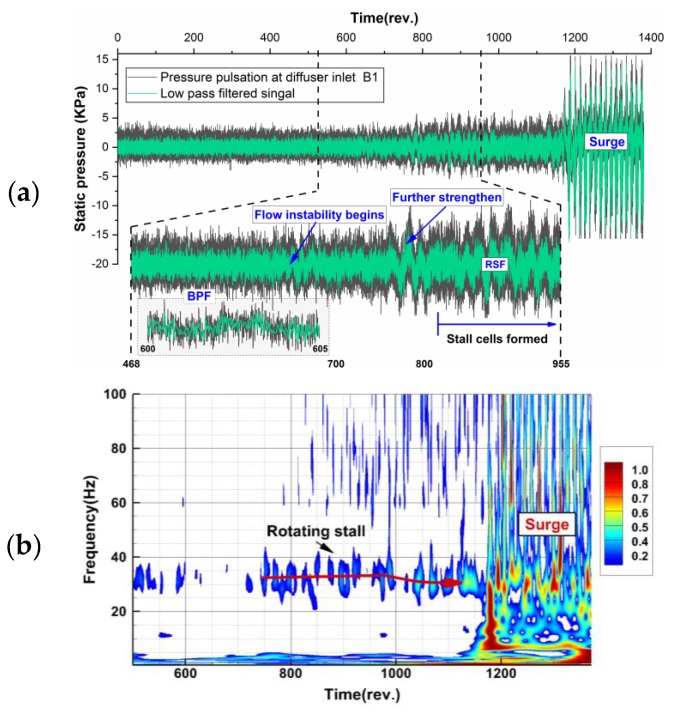
Rotating stall evolution and surge happened during throttling process at $100\%\Omega_{\mathrm{norm}}$: (**a**) pressure traces from 0 to 1370 revolutions; (**b**) time-frequency spectrum based on wavelet transform.

The raw pressure measurements and corresponding low pass filtered signal with the blade passing frequency $f_{\mathrm{BPF}}$ when entering surge are presented in Figure 7. This pressure signal is measured at the inlet of the diffuser passage. Before flow instability starts, the pressure fluctuating frequency is dominated by the jet-wake flow feature at the outlet of the impeller. During the throttling process, the pressure will become unstable and low-frequency variation with large amplitude starts to appear. This change in signal structure means that after these stall cells formed in the flow passage, the jet-wake flow pattern in stable operating condition will be superimposed by the stall dominated fluctuation. And, these stall cells will become stable and propagate in circumference after a short time. The static pressure measured during the throttling process is further analyzed by the wavelet transform. The scalogram values are scaled by the maximum absolute value at each level and frequencies are displayed on a linear scale. The flow behavior described above can also be viewed in time-frequency space. When the instability is further enhanced after 750 revolutions, a stable low-frequency mode occurs, which is consistent with the signal structure evolution in the time domain. If the flow rate is further reduced, the compression system will enter surge and large fluctuation of static pressure occurs.

### 4.2. Rotating Stall Induced Vibration Identification during Speed Ramp

**Figure 8:**
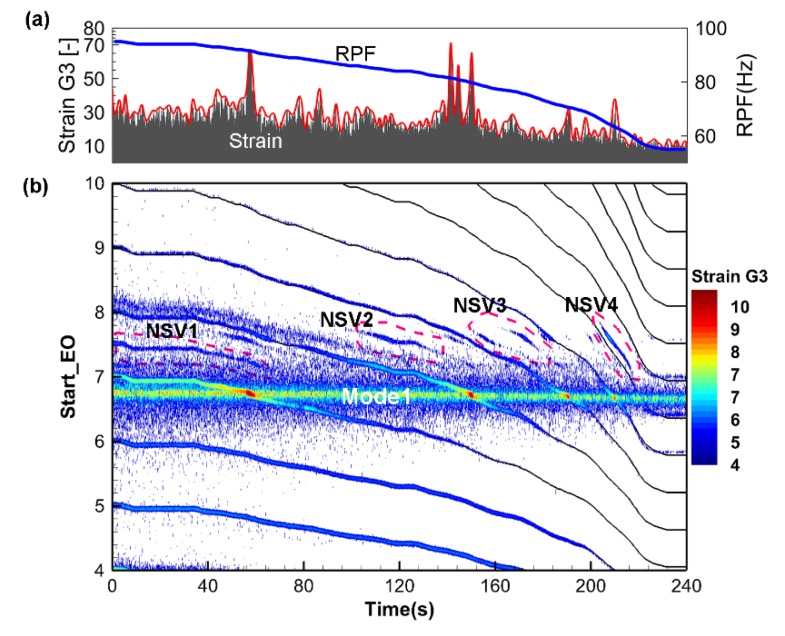
Blade vibration measured by Strain Gauge G3 during Speed Ramp: (**a**) time domain signal; (**b**) time-frequency spectrum based on STFT.

Different rotating stall induced vibration phenomenon is first found by speed ramp testing method. The centrifugal compressor is first set to run at $100\%\Omega_{\mathrm{norm}}$ speed and the mass flow is also adjusted to low flow rate to make the impeller disturbed by the rotating stall. Then, the shaft speed is continuously reduced to about $50\%\Omega_{\mathrm{norm}}$ speed after all the signal acquisition equipment is get prepared. Figure 8a gives the time signal of gauge G3. The envelope of the signal is calculated to show the interaction between impeller mode with engine order excitation. The rotating passing frequency (RPF) of the shaft is also plotted on the right axis. Figure 8b gives the entire short time Fourier transform (STFT) spectrum of the result. It should be mentioned that the frequency value is normalized by the shaft frequency at the beginning. The engine order number is displayed as the left *y*-axis. These integers are used to show different engine order lines when the speed is continuously reduced. The non-synchronous blade response information is mainly found around the mode 1 of the impeller. It is consistent with the rotating stall induced vibration frequency determined by Equation (2) since the stall cell number is limited. Four different non-integer exciting regions are found and denoted by the dashed line. However, compared to mode 1 response of the impeller, the rotating stall induced vibration is still not so apparent. It is mainly due to the transient and instability nature of rotating stall under variable speed operation condition. The stalled state will change according to impeller speed and mass flow rate. But the stall cell formed rotating aerodynamic force gives us a dangerous warning because it has the same or even larger energy level compared to synchronous exciting. Large amplitude vibrations will appear if the exciting frequency coincides with the impeller modes.

Although the variable speed testing method gives us a quick way to view the rotating stall induced vibration information. Due to the operation is transient and unstable, it is not suitable for stall parameters identification. In addition, in order to evaluate the blade vibration, quasi-steady operation under the rotating stall condition is also needed.

### 4.3. Stall Parameters Identification Based on Circumferential Pressure Pulsation Signals

**Figure 9:**
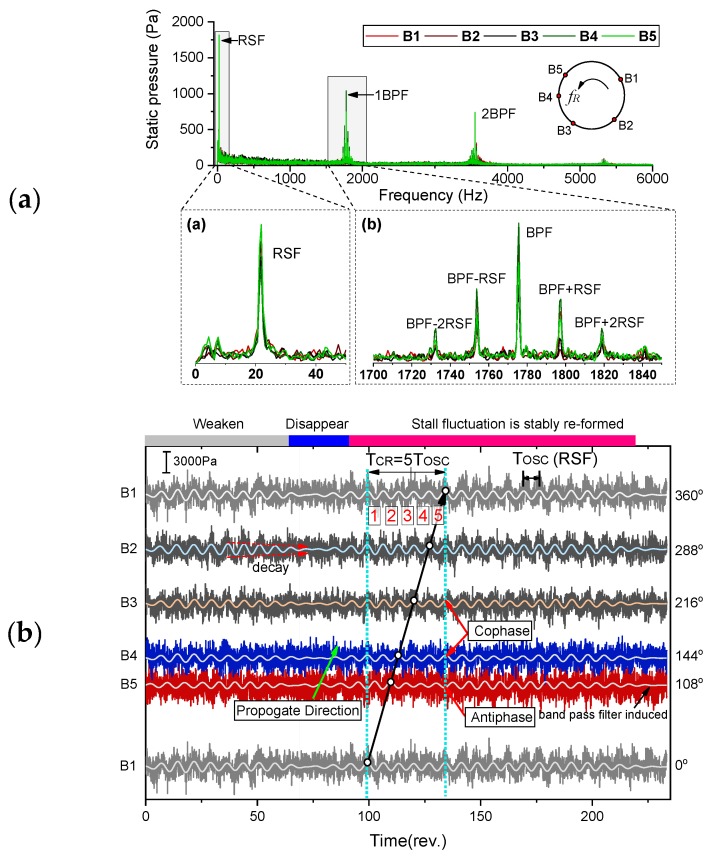
Rotating stall identification and parameter characterization. (**a**) FFT spectrum of diffuser inlet pressure signal; (**b**) spatial pressure distribution at stalled condition.

In order to characterize the stall cell pattern during different quasi-steady operating conditions, the FFT spectrum combined with circumferential pressure distribution is first used for manual identification of the stall parameters since it is the most used and reliable method for industrial application [28]. Pressure pulsation signal spectrum is first analyzed to identify stall occurrences. Figure 9a gives the frequency spectrum measured at diffuser inlet when the compressor operates at OP1 depicted in Figure 3. A significant low frequency of $f_{\mathrm{PC}}=21.9\;\mathrm{Hz}$ below the shaft frequency occurs, and there exists apparent signal modulation phenomenon on both sides of the BPF, which means the original rotational perturbation of the impeller is modulated by stall cell-induced fluctuation. In Figure 9b, a graphical matter based on bandpass filtered pressure traces is further used to acquire the stall parameters, including the number of stall cells ${\mathrm{NB}}_{C}$, rotating speed $f_{\mathrm{cell}}$ and propagation direction [26]. Within the graph, pressure signals are plotted from bottom to top according to their circumferential positions in direction of impeller rotation. In order to form one revolution, the pressure signal of the first sensor is plotted once more. As it can be viewed from the graph, the low-frequency disturbance is arising and decaying in different stable periods. However, it will also become weak and disappear for a short time but reformed immediately. The propagation direction is first analyzed based on the dynamic behavior of the pressure signals from closely distributed two sensors, B5 and B4. It can be clearly judged that the stall propagation direction is the same as the impeller rotation. During stall formed period, it can be found that the circumferential 5-channel band-pass filtered pressure signal is almost the same phase at any time, but the fourth channel is in the inverted state. According to the sensor distribution angles, this phenomenon indicates that 36° corresponds to half of the distance between two adjacent stall cells in a cylindrical coordinate system along the direction of propagation. The identified stall mode is a stall group consisting of five cells. If we connect a peak of B1-0° with the corresponds one of B5-108°, which is delayed one and a half cycle, and extend to P1-360°, the stall propagation trajectory which crosses all signal peak positions are extracted.

However, the pressure trace diagram method is a manual approach and can lead to some misunderstandings in some cases. The cross-correlation is further used to confirm the identified stall mode and further automate the identification process.

(16) $$R_{ij}\left[n\right]=\frac{1}{N}\sum_{k=1}^{N}P_{i}\left(k\right)P_{j}\left(k+n\right)$$

**Figure 10:**
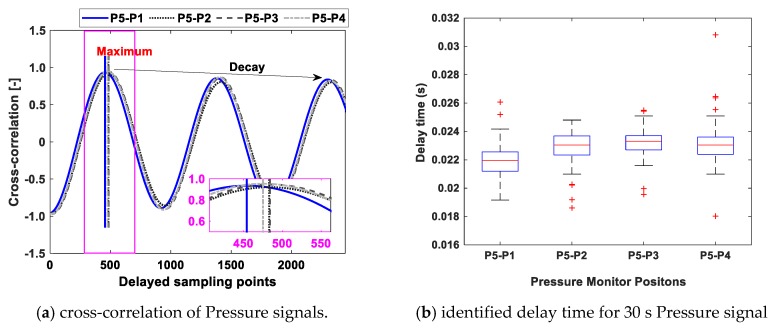
Stall mode identification and parameter characterization using cross-correlation analysis.

Equation (16) is the discrete form of the cross-correlation between signal $P_{i}$ and $P_{j}$. Figure 10a shows the cross-correlation results of the 5-channel pressure signals. Before cross-correlation, bandpass filtering is needed to extract the stall wave. The delay time can be calculated by the number of delayed sampling points corresponding to the local maximum of the cross-correlation. As can be seen, the delayed sampling points is around 450–500 which means the delay time is about 0.022–0.024 s based on the sampling frequency. In order to access the long-term behavior of the stall propagation, the delay time between stall cells is further calculated based on 30s’ pressure pulsation signal. Figure 10b gives the box plot of the result. This result means upon the stall cell is formed around the impeller it will not change and keep stable. Since the aerodynamic force is not changed, the induced vibration should also be stable which can be further proven in the following content.

**Figure 11:**
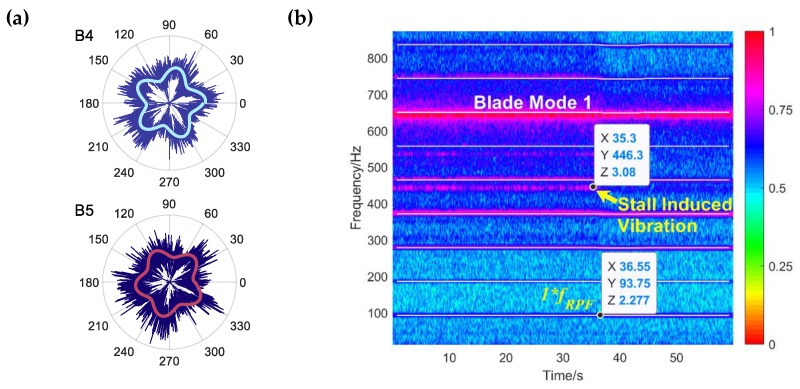
Rotating stall induced impeller blade vibration: (**a**) 5 stall cells dominated aerodynamic exciting force in polar coordinate and (**b**) time-frequency analysis of Strain Gauge G3.

First, the five stall cells induced pressure traces can be plotted in polar coordinate based on the identified stall parameters, as shown in Figure 11a. These circumferential distributions of the pressure wave give a much better description of the footprint of the rotating stall cells at different monitoring positions. Figure 11b gives the corresponding time-frequency spectrum of blade vibration. During the rotating stall period, the five stall cells mode induced blade vibration is first found. The engine order of the vibration is 4.77. It is not an integer of the shaft rotating passing frequency which means non-synchronous vibration happened during rotating stall operating condition. Based on the identified parameters and Equation (2), the relationship can be verified:

(17) $$\left\{ \begin{array}{l} f_{e}^{Strain}=4.773\cdot f_{\mathrm{shaft}}=446.3\;\mathrm{Hz}\\ f_{e}^{5cell}={\mathrm{NB}}_{C}\cdot f_{\mathrm{shaft}}-{\mathrm{NB}}_{C}\cdot f_{\mathrm{cell}}=445.625\;\mathrm{Hz}\\ f_{\mathrm{shaft}}=93.5\;\mathrm{Hz},\;\;{\mathrm{NB}}_{C}=5 \end{array}\right.$$

So, it has $f_{e}^{Strain}\approx f_{e}^{5cell}$, where $f_{e}^{Strain}$ is the vibration frequency existing in the blade response signal, $f_{e}^{5cell}$ is the predicted blade vibration frequency calculated by rotating stall parameters and Equation (2). $f_{\mathrm{cell}}$ is the rotating frequency of one individual stall cell and has $f_{\mathrm{cell}}=f_{\mathrm{PC}}/{\mathrm{NB}}_{C}$. As can be seen, these two frequencies agree quite well. There is only a small difference (less than 1 Hz) between $f_{e}^{Strain}$ and $f_{e}^{5cell}$ due to numerical error or spectral resolution. And the propagation direction is the same as the impeller rotation since ”−” is used in Equation (17) instead of ”+”. The method of judging the direction of propagation through the low-frequency component fluctuation curve is still not very robust. Under some circumstances, the direction information may lead to being misinterpreted or cannot be decided only based on the signal dynamic behaviors. For example, if B1-B3 signals are used, the direction is hard to be decided. As well, it will also cause the cell number be misidentified when the spatial resolution of sensors is very limited. Since the relationship between pressure pulsation and blade vibration provided by Equation (2) is proven by analytical models and experimental analysis. Considering the relationship between the vibration and pressure fluctuating not only contains the cell number information but also includes the propagation direction, the vibration information can be combined to give a more reliable result.

### 4.4. Vibration Quantification Based on Tip Timing Measurement

**Figure 12:**
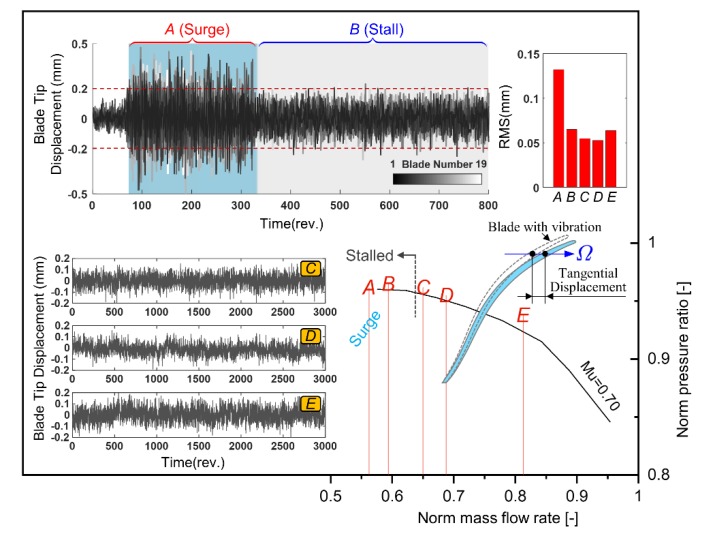
Blade tip timing results under five operating points with different mass flow rate varying from surge to choke.

The tip displacement signal is measured close to the leading edge of the impeller blade. Detailed monitoring position can be referred to Figure 6a. Based on the blade tip timing system, the tangential vibration of all blades under different operating points can be recorded for further displacement evaluation. Figure 12 gives the calculated blade tip displacement signal. The vibration behavior under low mass flow rate is concerned. For the operating point *A*, surge happens in this compression system and leads to an extremely large vibration amplitude. When the mass flow rate is adjusted during the experiment to make the compressor return to rotating stall condition (operating point *B*), the vibration is reduced significantly. Root mean square (RMS) level of the vibration signal is further calculated to show the influence of different mass flow rate. Results are plotted at the right top of Figure 12. From the perspective of the RMS value, the blade vibration is similar for operating point B to E. However, these small differences still show blade vibration trend and give a reasonable result of the vibration monitoring. The vibration is slightly reduced from operating point *B* to *D*, which corresponds to the mass flow change varying from stall to design point. However, when it comes to the choke boundary at operating point *E*, the vibration amplitude increases inversely.

It should also be mentioned that due to the arrangement of the BTT probes and the limited number of sensors, the frequency of the BTT signal cannot be accurately recovered. Non-intrusive detection of stall-vibration can be performed based on the tip timing signal in the future. The layout of the BTT probes and the experimental operating points of the compressor should be carefully designed and selected to capture this phenomenon from the perspective of aerodynamics and signal processing.

## 5. Conclusions

This paper focuses on the non-synchronous blade vibration at stall and near surge operating of the centrifugal compressor. A theoretical model was first built up to describe the impeller vibration. Under the traveling wave excitation, the forced response function of the simplified disc was deduced. The impeller resonance condition was further acquired based on the steady part of the response function. The relationship between stall wave and impeller vibration was also correlated using the parameters which describe the stall mode. The derived relationship is consistent with previous understanding. The stall excitation law of the rotating stall can be viewed from a novel aspect, and at the same time, it can be found that both the rotating stall and its higher harmonics can induce the blade vibration and resonance with corresponding nodal diameter modes.

Experimental work was further conducted based on the industrial compressor test rig using an unshrouded centrifugal impeller equipped with vaned diffuser. During experiments, multiple signals from casing-mounted pressure transducers, strain gauges, and tip timing sensors were simultaneously acquired to provide a detailed insight into this physical phenomenon. Both transient and quasi-steady operating of compressor were designed and tested in detail to reveal the underlying mechanism and verify the relationship between pressure pulsation and blade vibration. The experimental investigation included: (1) detection and parameter characterization of rotating stall cells. (2) clearly interpretation and relationship verification of non-synchronous blade vibration. (3) quantify the impact of stall cells on all blade response amplitude. The throttling test of the compressor shown the dynamical behavior of airflow and a stable low-frequency mode appeared as it approached the stall boundary. The rotating stall was first understood from aerodynamic perspective. Then, the fluid-structure interaction measurements were further conducted using varying speed operating of the compressor. With time-frequency analysis method, the blade non-synchronous vibration regions were quickly found. Further, the quasi-steady operating of compressor was selected and measured for a long time in order to stabilize the stall and identify the specific cells mode. Both circumferential pressure tracking and cross-correlation methods were used to give an appropriate result. The frequency spectrum and spatial distribution characteristics of the pressure signal were also correlated with the blade vibration, which also shown the correctness of the theoretical model. The quantified blade response provides realistic compressor representative vibration data, which is the basis for the aeroelastic design of the impeller blades considering rotating stall. The overall test method in this paper is also important for stall vibration and resonance-free design using experimental verification methods.

Stall cells are extra excitation sources and can cause blade forced vibration at a specific frequency, which should be considered for more reliable operating of the compressor. In addition, the stall cell number is the core parameter which mostly affects the vibration frequency and occurrence of blade resonance. The first-order impeller mode is quite close to the stall excitation frequency and requires more attention in the future design phase of the impeller structure. The theoretical and experimental work of this paper contributes to the basic understanding and quantification of compressor stall and vibration. Further investigations are required to non-intrusive detect and control the rotating cells.

## Figures and Tables

**Table 1:** Specifications of the investigated compression stage.

Parameters	Value
Number of inlet guide vanes	11
Number of impeller blades	19
Number of diffuser vanes	20
Number of return channel vanes	18
Impeller outlet diameter D2 (mm)	810
Impeller outlet width b2 (mm)	57.5
Diffuser inlet diameter D3 (mm)	900
Diffuser outlet diameter D4 (mm)	1242
